# Neurophysiological basis of respiratory discomfort improvement by mandibular advancement in awake OSA patients

**DOI:** 10.14814/phy2.15951

**Published:** 2024-02-19

**Authors:** Rémi Valentin, Marie‐Cécile Niérat, Nicolas Wattiez, Olivier Jacq, Maxens Decavèle, Isabelle Arnulf, Thomas Similowski, Valérie Attali

**Affiliations:** ^1^ INSERM, UMRS1158 Neurophysiologie Respiratoire Expérimentale et Clinique Sorbonne Université Paris France; ^2^ Hôpital Pitié‐Salpêtrière, Département R3S, Service des Pathologies du Sommeil (Département R3S) AP‐HP, Groupe Hospitalier Universitaire APHP‐Sorbonne Université Paris France; ^3^ Institut de Biomécanique Humaine Georges Charpak École Nationale Supérieure des Arts et Métiers Paris France; ^4^ Service de Médecine Intensive et Réanimation (Département R3S) Groupe Hospitalier Universitaire APHP‐Sorbonne Université Paris France; ^5^ Paris Brain Institute (ICM) Sorbonne Université Paris France; ^6^ Hôpital, Pitié‐Salpêtrière, Département R3S AP‐HP, Groupe Hospitalier APHP‐Sorbonne Université Paris France

**Keywords:** dyspnea, electroencephalography, neurophysiology, OSA, respiratory drive

## Abstract

Patients with obstructive sleep apneas (OSA) do not complain from dyspnea during resting breathing. Placement of a mandibular advancement device (MAD) can lead to a sense of improved respiratory comfort (“pseudo‐relief”) ascribed to a habituation phenomenon. To substantiate this conjecture, we hypothesized that, in non‐dyspneic awake OSA patients, respiratory‐related electroencephalographic figures, abnormally present during awake resting breathing, would disappear or change in parallel with MAD‐associated pseudo‐relief. In 20 patients, we compared natural breathing and breathing with MAD on: breathing discomfort (transitional visual analog scale, VAS‐2); upper airway mechanics, assessed in terms of pressure peak/time to peak (TTP) ratio respiratory‐related electroencephalography (EEG) signatures, including slow event‐related preinspiratory potentials; and a between‐state discrimination based on continuous connectivity evaluation. MAD improved breathing and upper airway mechanics. The 8 patients in whom the EEG between‐state discrimination was considered effective exhibited higher Peak/TTP improvement and transitional VAS ratings while wearing MAD than the 12 patients where it was not. These results support the notion of habituation to abnormal respiratory‐related afferents in OSA patients and fuel the causative nature of the relationship between dyspnea, respiratory‐related motor cortical activity and impaired upper airway mechanics in this setting.

## 1 INTRODUCTION

Patients suffering from obstructive sleep apneas (OSA) do not complain of dyspnea during awake resting breathing, notwithstanding abnormalities in upper airway mechanics representing an inspiratory load (Attali et al., 2019). Yet, despite this absence of respiratory complaints, the placement of a mandibular advancement device (MAD), known to improve upper airway mechanics (Gakwaya et al., 2014), can lead to a sense of improved respiratory comfort (Attali et al., 2019), hereafter termed “pseudo‐relief”. The intensity of this sensation correlates with the degree of mechanical improvement brought about by the MAD (Attali et al., 2019). This phenomenon has been interpreted as resulting from habituation to the respiratory sensations that would typically be associated with the respiratory‐related brain processing of OSA‐related abnormal respiratory afferents or the corresponding compensatory mechanisms. Indeed, OSA patients have an increased drive to breathe (Mezzanotte et al., 1992; Saboisky et al., 2007; Steier et al., 2010) and exhibit a respiratory‐related activation of the cerebral cortex visible under the form of slow preinspiratory potentials (PIPs) (Launois et al., 2015), two circumstances generally associated with dyspnea.

Respiratory habituation has been described experimentally (Subhan et al., 2003; Von Leupoldt et al., 2011; Wan et al., 2009) and hypothesized to proceed from downregulation of the insular cortex (Stoeckel et al., 2015; Von Leupoldt et al., 2011) or impaired somatosensory processing (Davenport et al., 2000; Fauroux et al., 2007) as it exists in OSA patients (Donzel‐Raynaud et al., 2009; Grippo et al., 2011). Demonstrating respiratory habituation in OSA patients would require showing first that a neurophysiological phenomenon usually associated with dyspnea can be evidenced in the absence of dyspnea and second that this phenomenon disappears in response to an intervention associated with “pseudo‐relief”.

Based on the presence of PIPs in certain patients with OSA (Launois et al., 2015) and on the association of PIPs with dyspnea in certain clinical contexts like amyotrophic lateral sclerosis (Georges et al., 2016) or mechanical ventilation in critically ill patients (Decavèle et al., 2023; Raux et al., 2019), we hypothesized that MAD‐associated respiratory “pseudo‐relief” would relate with MAD‐associated changes in respiratory‐related cortical activity.

We assessed the electroencephalography (EEG) activity by using two techniques: (1) detection of preinspiratory activity (PIP) and (2) an EEG classifier approach that assess global connectivity changes (Grosselin et al., 2018; Hudson et al., 2016; Navarro‐Sune et al., 2017). In patients under mechanical ventilation an area under the receiver operating characteristic curve (ROC) area under the curve (AUC) of 0.7 or more on the EEG classifier, was associated with the convergent relief of dyspnea and disappearance of PIPs after optimization of ventilatory parameters (Raux et al., 2019).

## 2 MATERIALS AND METHODS

### 2.1 Participants

We included 20 patients (4 women, median age 58 years [49–66], a body mass index (BMI) 26 kg/m^2^ [24–31]). Patients with a BMI ≥ 35 were excluded of study. They had no history of neurological or other respiratory disorders, no intake of psychotropic medications and no previous UA surgery. All had moderate to severe OSA characterized by an apnea hypopnea index (AHI) of 32 [28–48] events/h and a score at the Epworth somnolence scale (Epworth sleepiness scale, ESS; 0–24) before treatment of 11 [9–15]. All were treated for 3 months or more by a custom‐made MAD (Narval CC®, Resmed Ltd, France) titrated by a dental specialist. The MAD treatment was considered as optimal, based on symptoms control on ESS: 6 [5–7] (reduction of 6 [3–8] *p* < 0.001) and residual AHI 6 [3–14] (reduction of 23 [16–30]; *p* < 0.001). This study was conducted after legal and ethical approval by the *Comité de Protection des Personnes Ile‐de‐France VI—Pitié‐Salpêtrière (ID RCB: 2013‐A00158‐37)*. Participants received information on the purpose and procedures of this study and provided written consent to participate.

### 2.2 Experimental protocol

Participants were instructed to stop their MAD treatment 10 days before this experimental evaluation. The whole experiment was done while awake. Patients were sitting down on an inclinable chair, in a 45 degrees semi‐recumbent position, legs in decline position and with a cervical collar maintaining neck in neutral position. Once settled in a comfortable position, patients were instructed to score their breathing sensations on a visual analog scale (VAS) (see below).

We equipped them with a 32 electrodes EEG cap (Acticap, Brain Products, Germany) connected to an EEG preamplifier (V‐Amp, Brain Products, Germany) from which the signals were digitized at 2000 Hz. Simultaneously, a nasal canula recorded ventilatory signal by differential pressure transduction (DP15 Pressure Sensor ADInstruments, NZ), digitalized at 200 Hz (Powerlab 16/30, ADInstruments, NZ).

We recorded two breathing conditions, each lasting 20 min, one without MAD (natural breathing), the second while wearing the MAD.

### 2.3 Breathing discomfort

Before EEG acquisition, patients were asked to answer the trigger question “do you feel breathing discomfort (yes/no)” at rest. Independently from the answer to this trigger question they were asked to rate their breathing discomfort using two VASs under natural breathing and MAD conditions. The VAS‐1 assessed breathing discomfort on a nongraduated 100 mm visual analog scale anchored by “no breathing discomfort” at the left end and “intolerable breathing discomfort” at the right end (results expressed in % full scale). The VAS‐2 consisted on a “transitional” evaluation of the change in breathing discomfort before and after wearing MAD, on a nongraduated 100 mm scale anchored from “extreme deterioration” at the left end to “extreme improvement” on the right end, with a middle marker to indicate “no change” (results expressed in % of middle marker to extremes with “+” sign for improvement and “−” sign for deterioration) (Attali et al., 2019).

### 2.4 Breathing pattern

**FIGURE 1:**
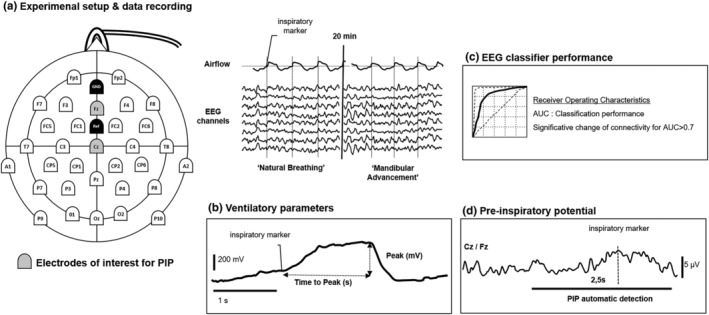
Schematic representation of the experimental setup and data analysis process. (a) Electroencephalography (EEG) was recorded using a 32 electrodes cap synchronized with nasal pressure sensor. (b) Each breathing cycle was marked at local maximum pressure amplitude (Peak) to calculate time to peak (TTP) from inspiratory onset. Peak/TTP ratio was calculated to appreciate a modification of breathing cycle time and amplitude. (c) Modification of EEG connectivity was assessed by area under the curve (AUC) of EEG classifier based on Riemannian geometry. An AUC >0.7 was considered as significative modification of EEG activity. (d) EEG was segmented in inspiratory epochs and were averaged after artifact rejection. Algorithm detected preinspiratory potentials (PIP) within 1.5 s before inspiratory onset.

The breathing pattern was extracted from the nasal pressure signal and inspiratory onsets were marked using an automatic trigger, visual artifacts were rejected manually (Figure 1a). None participant had predominant/permanent mouth breathing. The breathing mode (nasal or mouth) was monitored visually and on the nasal pressure signal; artifacts due to intermittent mouth breathing, were removed during analysis. We assessed peak pressure at inspiration (Peak) defined by local maximum of negative pressure between two inspiratory onsets and time to peak (TTP) defined by the time from inspiration onset to maximum pressure peak. Peak pressure at inspiration reflects ventilation maximum flow and should increase if UA critical closing pressure was improved by mandibular advancement. TTP as an estimation of inspiratory time should decrease with the reduction of obstruction while wearing MAD (Voskrebenzev et al., 2018). Therefore, composite parameter Peak/TTP should increase whether mandibular advancement would improve breathing signal amplitude (Peak) or slope (TTP) (Figure 1b).

### 2.5 Respiratory‐related cortical activity

#### 2.5.1 Riemannian EEG classifier

As described in details elsewhere (Grosselin et al., 2018; Hudson et al., 2016; Navarro‐Sune et al., 2017), an EEG classifier based on Riemannian geometry was used to compare EEG recordings and estimate the performance of the classification to detect a modification in EEG signature—which is the reflection of a change in brain state—between two conditions (Grosselin et al., 2018; Hudson et al., 2016; Navarro‐Sune et al., 2017; Raux et al., 2019). The classifier was first trained on natural breathing EEG matrices serving as reference to perform classification. Then the classifier compared EEG covariance matrices obtained during breathing with MAD and calculated the average deviation from the natural breathing distribution to estimate the rejection threshold indicating a significant change in cortical activity (Mason & Graham, 2002). Classification performance was evaluated by a cross‐validation process: the “natural breathing” period was divided into 10 equal EEG segments and comparisons between 9 of these segments and the data of the “MAD” condition were repeated 9 times to take all combinations into account. The results were plotted as ROC curves and the prediction AUC of 1 and 0.5 indicated perfected and random discrimination, respectively (Figure 1c). An AUC ≥0.7 outcome was considered as satisfactory to identify a cortical activity modification (Raux et al., 2019; Taytard et al., 2022).

#### 2.5.2 Preinspiratory potentials

According to the method previously described inspiratory time‐locked segments were averaged for both conditions (Georges et al., 2016; Launois et al., 2015; Nguyen et al., 2018; Raux et al., 2007; Tremoureux, Raux, Hudson, et al., 2014). Before data treatment, we used the inspiratory markers for EEG segmentation from −2.5 s before each inspiratory marker to 1 s after. Data was resampled to 250 Hz. We used semi‐automatized artifact rejection (EEG gradient ≥5 μV/ms; EEG amplitude ≥50 μV) and visual validation to preprocess EEG data. An in‐house computational algorithm based on time window and amplitude of EEG signal preceding inspiratory onset identified presence (PIP+) or absence (PIP−) of PIP either on Cz or Fz electrodes using Matlab (Mathworks Inc, USA) (Figure 1d). PIP+ Patients during natural breathing were retained to identify PIP modification profiles: PIP− while wearing MAD (PIP correction) and PIP+ while wearing MAD (PIP persistence).

### 2.6 Statistical analysis

**FIGURE 2:**
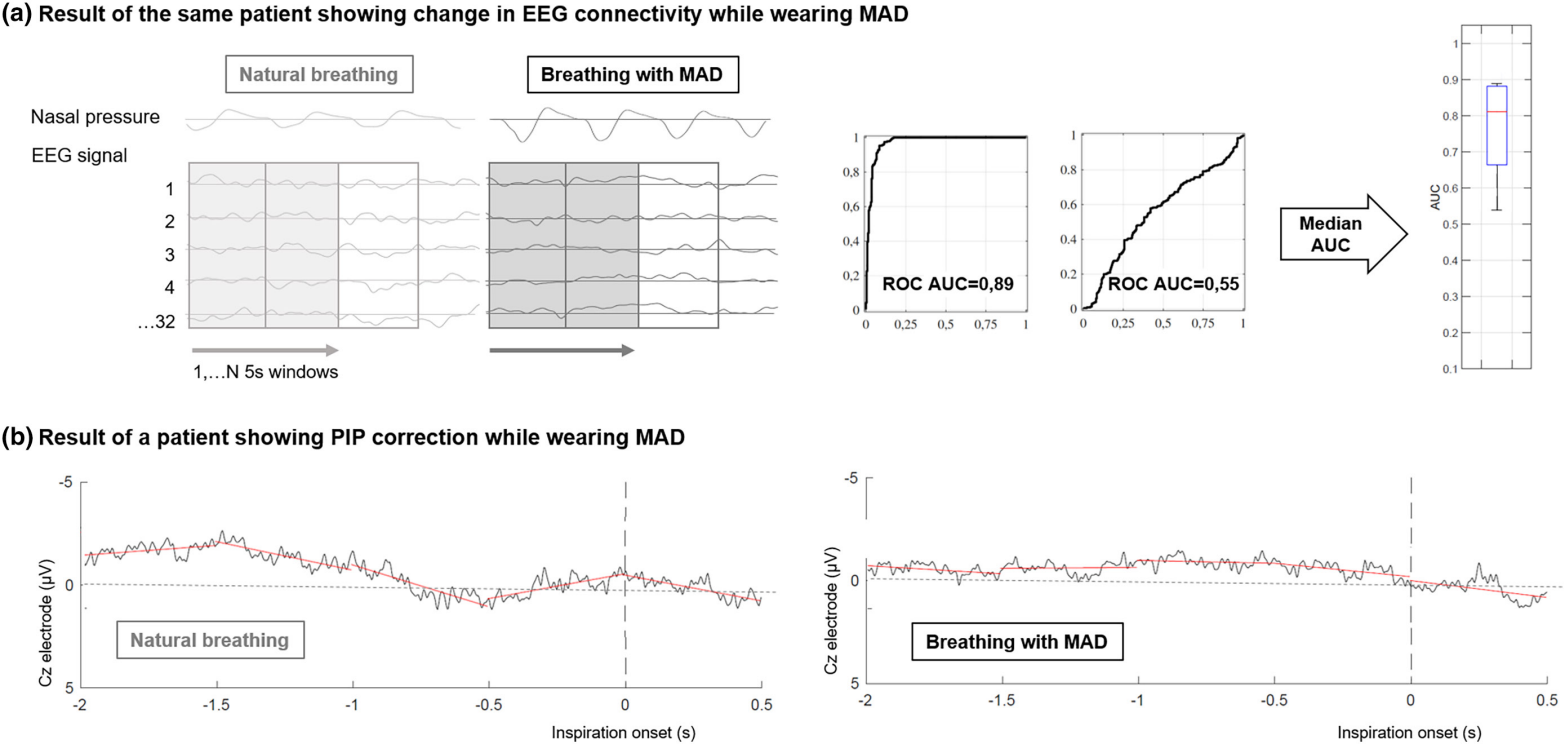
Results of one patient regarding preinspiratory potentials (PIP) detection and electroencephalography (EEG) classifier. (a) Average inspiratory epochs on Cz and Fz electrodes were divided in five windows of 0.5 s (red lines), preinspiratory activity was identified by automatic detection of amplitude and latency before inspiratory onset. During natural breathing, the presence of PIP for this patient is characterized by the slow negative shift around −0.5 s which cannot be found while breathing with mandibular advancement device (MAD). (b) On the left, covariance matrices are computed from electrode1 to 32 signals and 1 to N 5 s windows to capture the spatiotemporal dynamics of the EEG between natural breathing and breathing with MAD. In the middle, covariance matrices based on the Riemannian distance between EEG signals of both conditions was used to evaluate the ability of the classifier to detect a change in brain state using the receiver operating characteristics curve (ROC). On the right, a boxplot depicts the performance of the classifier to separate the two conditions in terms of area under the curve (AUC) (the box delineates the interquartile range of the AUC centered on median value, the whiskers correspond to the extreme values).

All statistics analyses were performed using Prism 9 © (GraphPad Software, USA). As all variables did not follow a normal distribution, the data was summarized in terms of their median and interquartile interval. Comparison of clinical features and breathing parameters between natural breathing and breathing with MAD was realized using Wilcoxon signed rank tests for paired data. Congruence between EEG classification and PIP correction after wearing MAD was assessed by Fischer's exact test. Then, patients with an AUC ≥0.7 on the EEG classifier were compared to patients with an AUC <0.7 on relative change in Peak (% of change), TTP (% change), Peak/TTP (% of change), VAS‐2 (% of scale), using a Mann–Whitney test for unpaired samples. In addition, we performed a sensitivity analysis in patients exhibiting a PIP+ during natural breathing and in whom the two EEG index provided congruent results, namely either PIP disappearance when breathing with MAD associated with a Riemannian classifier AUC ≥0.7 or PIP persistence when wearing the MAD associated with a Riemannian classifier AUC <0.7. These two subgroups were compared in terms of MAD‐associated changes in Peak (% of change), TTP (% change), Peak/TTP (% of change) and VAS‐2 (% of scale), using a Mann–Whitney test for unpaired samples. Differences were considered statistically significant for *p* values below 0.05 (Figure 2).

## 3 RESULTS

### 3.1 Breathing discomfort

None of the participants spontaneously responded «yes» to the trigger question “do you feel breathing discomfort?” at inclusion. Nevertheless, the VAS‐1 scale median score was 3 [0%–12%] during natural breathing and 0 [0%–5%] with MAD (−2 [−3.5 to 0], *p* = 0.011). The MAD‐associated change in breathing comfort on the VAS‐2 transitional scale was 26 [2%–56%], with 11 patients out of 20 reporting change over 20%.

### 3.2 Breathing pattern

Compared to natural breathing, breathing with MAD was associated with significantly higher values of Peak amplitude (from 14 [10–28] to 17 [10–37] mV, *p* = 0.002) and Peak/TTP ratio (from 31 [15–45] to 34 [14–57] mV/s *p* = 0,006) There was no significant change in TTP (0.7 [0.6–1] to 0.8 [0.6–1] s, *p* = 0.29) or breathing frequency (14 [12–18] to 14 [11–18] cycles/min, *p* = 0.43).

### 3.3 Respiratory‐related cortical activity

#### 3.3.1 Riemannian EEG classifier

**FIGURE 3:**
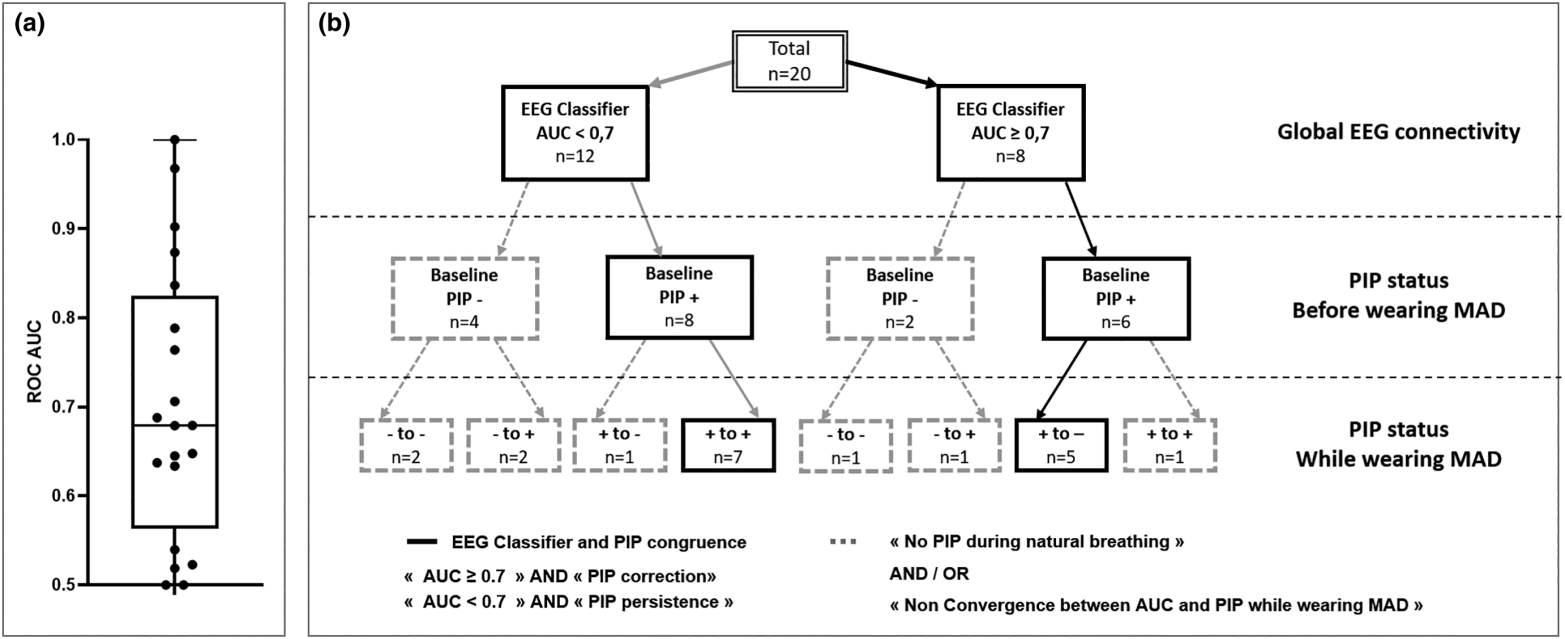
Electroencephalography (EEG) classifier area under the curve (AUC) and congruence with preinspiratory potential (PIP) between rest breathing and while wearing mandibular advancement device (MAD). (a) Boxplot centered on median with interquartile range, whiskers represent maximum and minimum values of EEG classification performance: receiver operating characteristics curve (ROC) area under the curve (AUC). An AUC ≥0.7 was considered as significative for a modification of EEG activity. (b) After EEG classification (AUC ≥0.7 in black and AUC <0.7 in gray), considering (PIP+) presence or (PIP−) absence at baseline rest condition, we described various profiles of PIP modification while wearing MAD (−to−, −to+, +to−, +to+). Patients with baseline PIP and PIP modification congruent with EEG classification profile («AUC <0.7/PIP+ to +» and «AUC ≥0.7/PIP+ to −» in plain line) were retained for further analysis.

We observed a change in cortical connectivity between natural breathing and MAD conditions with a median AUC on the EEG classifier of 0.68 [0.61–0.80] and an AUC ≥0.7 for eight patients (40%) (Figure 3a).

#### 3.3.2 Preinspiratory cortical activity

Fourteen patients (70%) exhibited PIPs during natural breathing (of which six had an AUC ≥0.7) (Figure 3b), which disappeared when wearing MAD in six cases (P+ to −, PIP correction: of which five had an AUC ≥0.7) (Figure 3b) and persisted in the eight remaining cases (P+ to +, PIP persistence: of which one had an AUC ≥0.7) (Figure 3b). PIP modification was congruent with global connectivity (AUC <0.7 and P+ to +; *n* = 7 OR AUC ≥0.7 and P+ to −; *n* = 5) for 12 of the 14 patients exhibiting PIP during normal breathing (patients in plain line on Figure 3a) (*p* = 0,025).

#### 3.3.3 Comparison of patients with AUC ≥0.7 and AUC <0.7 on EEG classifier

**FIGURE 4:**
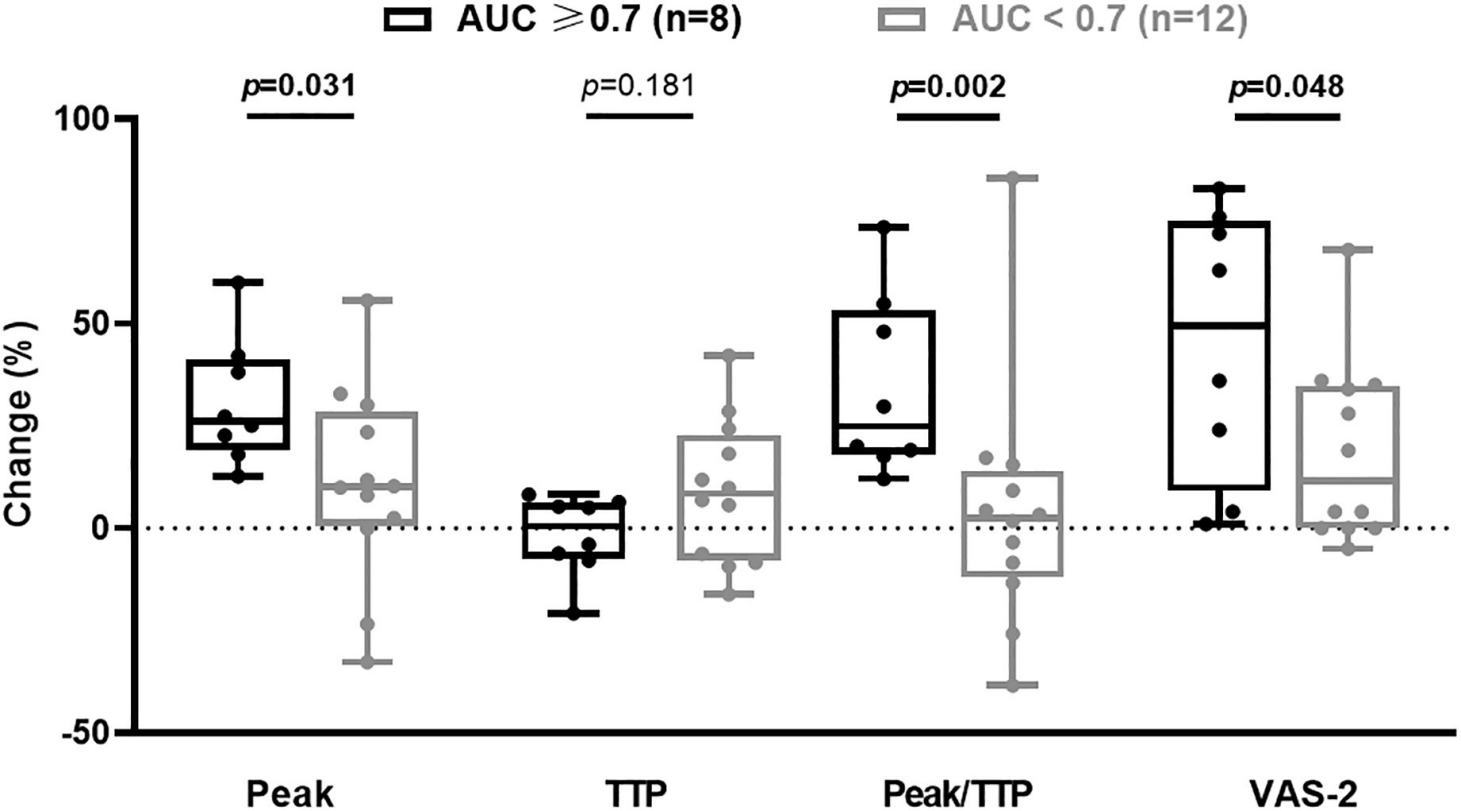
Relative change in breathing parameters while wearing mandibular advancement device (MAD) considering Electroencephalography (EEG) classifier and preinspiratory potential modification. In order to compare the ability of EEG classifier to identify patients with modification of breathing parameters while wearing MAD, we realized comparison for relative change in breathing parameters between 8 «area under the curve (AUC) ≥0.7» (in black) versus 12 «AUC <0.7» (in gray) participants. Percentage of change (% change) was calculated as the relative difference between baseline and while wearing MAD. Peak was the average local maximum of inspiratory pressure curve; time to peak (TTP) was the average time between inspiratory onset and local maximum of inspiratory pressure curve; Peak/TTP was the ratio reflecting inspiratory amplitude of pressure and inspiratory time; VAS‐2 was the score on transitional breathing discomfort scale before and after wearing MAD. Boxplot centered on median, interquartile range, whiskers represent maximum and minimum values.

Patients with AUC ≥0.7 (*n* = 8) and AUC <0.7 (*n* = 12) were comparable for age (58 [52–65] vs. 60 [49–66] years, *p* = 0.69), sex ratio (2/6 vs. 2/10 female/male ratio, *p* = 1) and BMI (28 [26–31] vs. 26 [24–28] kg/m^2^, *p* = 0.38). Patients with AUC ≥0.7 had higher dyspnea VAS‐2 transitional score (49 [9%–75%]) than patients with AUC <0.7 (11 [0%–35%]; *p* = 0.03). We also observed higher changes in Peak (26 [19%–41%] vs. 10 [0%–28%]; *p* = 0.03) and Peak/TTP (25 [18%–53%] vs. 3 [−12% to 14%]; *p* = 0.002) than patients with AUC <0.7 (Figure 4).

#### 3.3.4 Sensitivity analysis based on PIP and EEG classifier congruence

**FIGURE 5:**
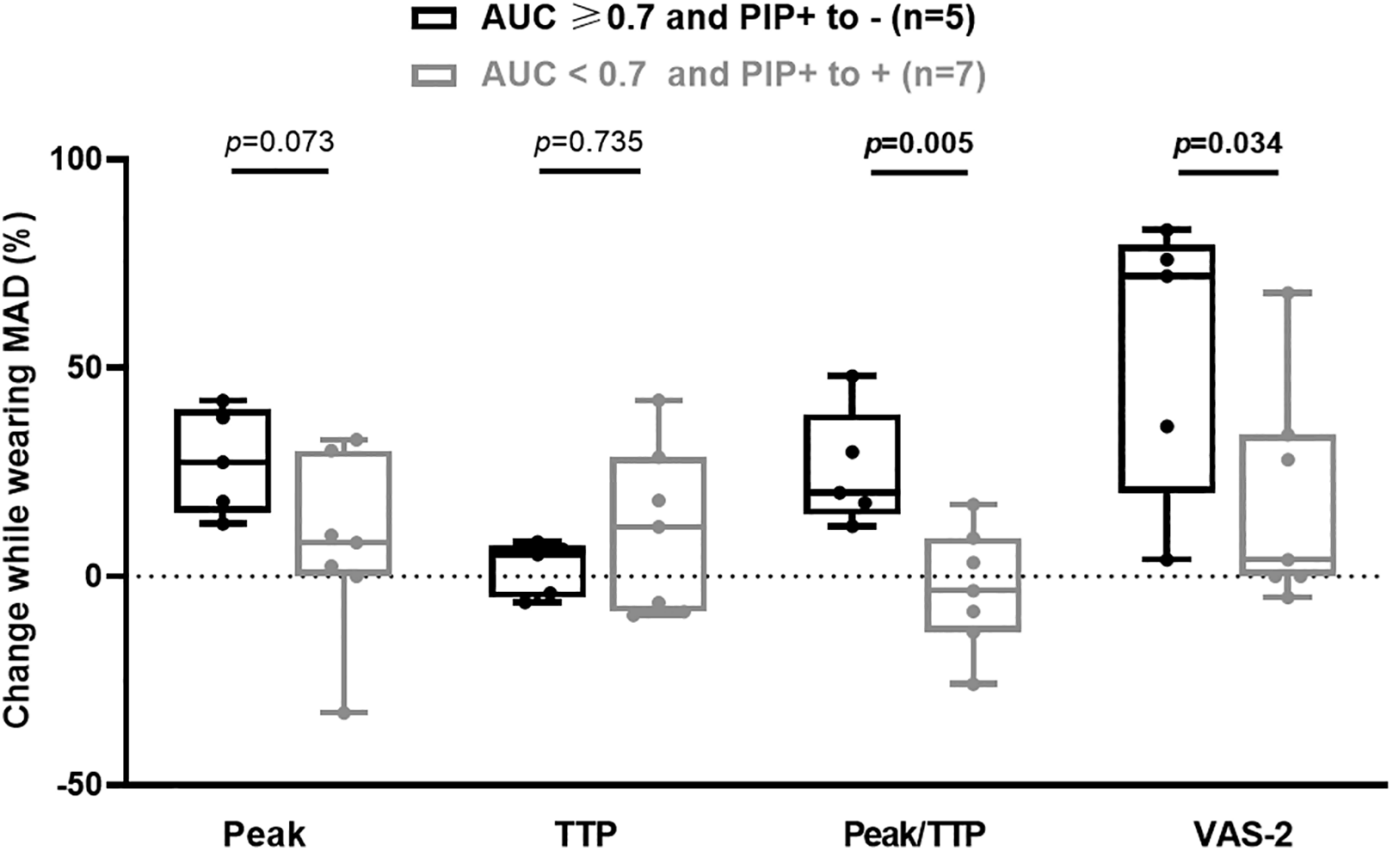
Relative change in breathing parameters while wearing mandibular advancement device (MAD) within preinspiratory potential (PIP)+ patients during rest breathing, considering electroencephalography (EEG) classifier and PIP modification. Patients exhibiting PIP during rest breathing were classified regarding both EEG classifier performance [area under the curve (AUC) ≥0.7] and modification of PIP while wearing MAD. In order to compare the ability of EEG classifier to identify patients with modification of breathing parameters while wearing MAD, we realized comparison for relative change in breathing parameters between 5 «AUC ≥0.7/PIP+ to −» (in black) versus 7 «AUC <0.7 and PIP+ to +» (in gray) participants. Percentage of change (% change) was calculated as the relative difference between baseline and while wearing MAD. Peak was the average local maximum of inspiratory pressure curve; time to peak (TTP) was the average time between inspiratory onset and local maximum of inspiratory pressure curve; Peak/TTP was the ratio reflecting inspiratory amplitude of pressure and inspiratory time; VAS‐2 was the score on transitional breathing discomfort scale before and after wearing MAD. Boxplot centered on median, interquartile range, whiskers represent maximum and minimum values.

The comparison between five patients congruently showing PIP correction while breathing with MAD and an AUC ≥0.7 (among the 8 AUC ≥0.7 patients, Figure 3b) and seven patients congruently showing PIP persistence while breathing with MAD and an AUC <0.7 (among the 12 AUC <0.7 patients, Figure 3b). The comparison of natural breathing and breathing when wearing the MAD showed significantly higher changes regarding Peak/TTP (20 [15%–39%] vs. −3 [−13% to 9%] change, *p* = 0.005) and dyspnea VAS‐2 transitional score (72 [20%–80%] vs. 4 [0%–34%], *p* = 0.034) for the “AUC ≥0.7 and PIP correction” group (Figure 5).

## 4 DISCUSSION

In line with our hypothesis, this study shows that patients with OSA who do not complain from dyspnea at rest and who report the largest pseudo‐relief in response to mandibular advancement exhibit concomitant EEG changes similar to the changes previously reported in response to dyspnea‐relieving interventions, for example after adjustments of ventilator settings in dyspneic mechanically ventilated critically ill patients (Raux et al., 2019). This provides a clear mechanistic argument in favor of habituation as the explanation of the absence of dyspnea in spite of abnormal respiratory mechanics OSA patients. This is also an additional argument for the causative nature of respiratory‐related EEG abnormalities in the pathogenesis of dyspnea (Decavèle et al., 2023; Georges et al., 2016; Raux et al., 2019).

### 4.1 General notions about the EEG approaches used

The two EEG approaches that we used to compare the natural breathing and the MAD breathing differ in their principle. The EEG classifier based on Riemannian geometry provides a global cortical connectivity analysis (i.e. including motor, premotor, somatosensory cortical areas and deep cortical areas) without specific reference to breathing. Interpreting an EEG change detected by this technique as respiratory in origin can only result from a contextual analysis. In the present case, natural breathing and MAD breathing differed in terms of respiratory mechanics and breathing comfort, which makes it reasonable to assign the EEG connectivity changes identified to a respiratory origin. On the other hand, the PIP approach focuses on time‐locked, cortical preparation of inspiration (Macefield & Gandevia, 1991). The presence of PIPs is interpreted as bearing witness to an increased drive to breathe involving the supplementary motor area (SMA) (Raux et al., 2007) in addition to the baseline tonic activity of SMA (Laviolette et al., 2013) and the automatic control originating from the brainstem (Del Negro et al., 2018). PIPs are generally not observed in healthy subjects during natural breathing outside voluntary inspiration or speech (Tremoureux, Raux, Ranohavimparany, et al., 2014). They are therefore considered as the sign of a cortico‐subcortical “cooperation” to maintain ventilation in the presence of a defective automatic breathing control (Fink, 1961), and of respiratory loading in healthy subjects (Raux et al., 2007) and in patients with respiratory disease (Georges et al., 2016; Nguyen et al., 2018; Tremoureux, Raux, Hudson, et al., 2014). The presence of PIPs, pointing at activation of the SMA, has been associated with dyspnea in certain circumstances (Georges et al., 2016; Hudson et al., 2016; Morawiec et al., 2015; Raux et al., 2019). Yet dyspnea occurs concomitantly with the activation of a much larger brain network, involving projections to the sensory cortex and integration in the limbic cortex (Farb et al., 2013) including cingulate cortex (Straus et al., 1997), posterior insula (Farb et al., 2013), and connection between posterior insula and thalamus (Farb et al., 2013). This should make the EEG classifier approach sensitive for the detection of dyspnea modulation (Raux et al., 2019). In our patients, this is supported by the association of VAS‐2 score with the EEG classifier performances.

### 4.2 Significance of results

In this series, the detection of PIPs in 14 out of 20 patients confirms our previous observations in awake OSA patients (Launois et al., 2015, 2020). The presence of PIP suggests a wake‐related cortical adaptation to the intrinsic load at UA level, to improve the stabilizing activity of the pharyngeal dilator muscles and prevent obstructive events (Launois et al., 2015, 2020). MAD‐associated changes in PIPs and EEG classifier performances were congruent for 12 of the 14 PIP+ patients (86%) and for 15 patients in all patients (75%). In the five noncongruent patients, we observed a PIP modification in spite of an AUC <0.7 in three patients and an AUC ≥0.7 without PIP modification in two patients. PIP modification while AUC <0.7 could be explained by difference of nature between PIP analysis (amplitude analysis centered premotor cortical area and time‐locked on inspiration onset) (Georges et al., 2016; Launois et al., 2015; Nguyen et al., 2018; Raux et al., 2007; Tremoureux, Raux, Hudson, et al., 2014), and EEG classifier (covariance analysis of global connectivity) (Grosselin et al., 2018; Hudson et al., 2016; Navarro‐Sune et al., 2017; Raux et al., 2019). Therefore, a modification of PIP amplitude solely located around premotor areas could be less detectable by the classifier approach than low amplitude changes occurring in several cortical areas. Similarly, AUC ≥0.7 without PIP modification can be explained by low decrease of PIP amplitude but significant change in synchrony of premotor preparation network for inspiration. PIP could thus be the “visible top” of the connectivity changes induced by MAD. For the sake of consistency, we performed a sensitivity analysis in patients in whom the two EEG approaches were congruent. We observed similar results between this conservative approach and the main analysis, namely an improvement in breathing pattern and breathing comfort in patients with AUC ≥0.7. This suggests that the EEG classifier approach alone is reliable to detect MAD‐associated changes in respiratory cortical activity. Of note, those of our patients who exhibited congruent EEG changes were those in whom the largest pseudo‐relief and the largest changes in respiratory pattern were observed.

### 4.3 Study limitations

We have included patients treated with MAD for 3 months or more, but to avoid a potential “residual” effect of MAD, patients were instructed to stop their MAD treatment 10 days before the evaluation. We showed a similar prevalence of PIP than in the study of Launois et al which included non‐treated patients (Launois et al., 2015). Of note, MAD is a symptomatic and not a curative treatment. Consequently, while awake when the patient is not wearing the MAD, the mechanical properties of their upper airways are not improved and this may induce a respiratory‐related cortical activity in the form of a PIP. We recognize that a longitudinal evaluation of the effect of a MAD treatment on cortical activity and “pseudo‐relief” constitute a perspective of this work.

We acknowledge that the absence of a control group implies a conservative interpretation of the results, however we are confident that our results support a relation between upper airway mechanics, breathing sensations and brain connectivity in patients with obstructive sleep apnea syndrome (OSAS). The present study was designed on the basis of two previous studies which had included controls. The first study showed that the prevalence of PPI was significantly higher in patients with OSAS (60% in severe OSAS and 30% in mild to moderate), than in the control group (less than 10%) (*p* = 0.0336) (Launois et al., 2015). The second study showed that only patients with OSA showed an improvement in respiratory comfort when wearing a MAD while awake, and that this improvement was related to the severity of the abnormalities in upper airway mechanics. (Attali et al., 2019). We acknowledge that insufficient technical sensitivity could explain the imperfect homogeneity of our observations among patients. This could also be due to undetected phenotypic heterogeneity among the participants to our study especially regarding age which is positively related to OSAS prevalence (Bixler et al., 1998). The choice to exclude patients with a BMI superior to 35 Kg/m^2^ was made to be consistent with previous studies (Attali et al., 2019; Launois et al., 2015). We also acknowledge that further work is needed to better characterize the brain changes associated with MAD in patients with OSA of which the EEG is only a partial reflection. For example, functional resonance imaging would be necessary to determine whether the pseudo‐relief observed in our patient only proceeds from the mechanical improvement‐related suppression of the compensatory cortical activation (as suggested by the experimental study of MAD in inspiratory loading by Hashimoto et al. (2006), or if it also involves the activation of the specific dyspnea‐relief network described by Peiffer et al. (2008). Finally, although our study does bring a mechanistic argument for habituation as the explanation of the absence of dyspneic complaints in patients with OSA, it does not elucidate the mechanisms of this habituation. It would be interesting to confront the EEG responses to MAD to the alterations in somatosensory processing of respiratory stimuli that exists in OSA patients (Donzel‐Raynaud et al., 2009) and that could contribute to blunting respiratory sensations.

## 5 CONCLUSIONS AND PERSPECTIVES

There is emerging evidence that inspiratory loading is concurrent not only with respiratory‐related cortical activation, but also with impaired cognitive performances (Taytard et al., 2022). Interferences between cognition and respiratory‐related cortical activity has also been described in the absence of dyspnea in patients with congenital central alveolar hypoventilation (CCAH) (Sharman et al., 2014; Taytard et al., 2023) who are known to rely on their cerebral cortex to maintain ventilation during wakefulness in spite of a defective breathing automaticity (Tremoureux, Raux, Hudson, et al., 2014). Respiratory‐related cortical activation in patients with OSA could contribute to OSAS‐associated impairment of cognition (Jordan et al., 2014) and of other centrally modulated functions such a balance and gait (Clavel et al., 2020; Degache et al., 2016). In the present study not only do we confirm that respiratory‐related cortical activity is a reality in OSA patients, but we also show that this activity can be suppressed by as simple a mean as MAD. Therefore, our study justifies to assess the immediate cognitive effects of MAD (or any other means to suppress respiratory‐related cortical activity) in OSA, in addition to the more classically studied cognitive effects of the long‐term treatment of OSA.

## AUTHOR CONTRIBUTIONS

RV, MCN, OJ, MD and VA contributed to design and conception of the work, acquisition, analysis or interpretation of data, NW, IA and TS contributed to design of the work, analysis and interpretation of the data. All authors contributed to drafting and revisiting of the manuscript. All authors approved final version of the manuscript and all persons who qualify as author are listed.

## FUNDING INFORMATION

VA is the recipient of a grant “poste d'accueil APHP/Arts et Métiers ParisTech”, Délégation à la Recherche Clinique et à l'Innovation (DRCI), Assistance Publique Hôpitaux de Paris (APHP). RV was supported by a grant « Inter Carnot », Institut Carnot for his PhD doctorate.

## CONFLICT OF INTEREST STATEMENT

RV, MCN, NW, OJ declared no link of interest. MD reported no link of interest related to the study. Outside the submitted work he reported congress registration fees from ISIS Medical. IA, reported no link of interest related to the study. Outside the submitted work she took part in an advisory board of IDORSIA in 2020. TS reported no link of interest related to the study. Outside the submitted work he reported personal fees for consulting and teaching activities from ADEP Assistance, AstraZeneca France, Chiesi France, KPL consulting, Lungpacer Inc., OSO‐AI, TEVA France, Vitalaire, all outside the submitted work. VA had no link of interest related to the study. Outside the study, she took part in an advisory board of BIOPROJET in 2021.

## ETHICS STATEMENT

This study was conducted after legal and ethical approval by the Comité de Protection des Personnes Ile‐de‐France VI – Pitié‐Salpêtrière (ID RCB: 2013‐A00158‐37). All methods were conducted in strict adherence to the relevant guidelines and the Code of Ethics of the World Medical Association (Declaration of Helsinki). Prior to participation, the study subjects provided written informed consent.

## Data Availability

All original data from which graphical and/or tabular summary data is generated is archived and fully available to The Journal upon reasonable request.
