# Correction to “Neurophysiological basis of respiratory discomfort improvement by mandibular advancement in awake OSA patients”

**DOI:** 10.14814/phy2.16167

**Published:** 2024-07-24

Valentin, R., Niérat, M.‐C., Wattiez, N., Jacq, O., Decavèle, M., Arnulf, I., Similowski, T., & Attali, V. (2024). Neurophysiological basis of respiratory discomfort improvement by mandibular advancement in awake OSA patients. Physiological Reports, 12, e15951. https://doi.org/10.14814/phy2.15951.

The “Funding Information” section included text that was incorrect:

“VA is the recipient of a grant “poste d'accueil APHP/Arts et Métiers ParisTech”, Délégation à la Recherche Clinique et à l'Innovation (DRCI), Assistance Publique Hôpitaux de Paris (APHP). RV was supported by a grant “Inter Carnot”, Institut Carnot for his PhD doctorate.”

This should have read:

“VA is the recipient of a grant “contrat d'interface pour hospitalier”, Institut National de la Santé et de la Recherche Médicale (INSERM). RV was supported by a grant “projet inter Carnot” from Institut Carnot APHP (Assistance Publique‐Hôpitaux de Paris) and Institut Carnot ARTS (Actions de Recherche pour la Technologie et la Société) for his PhD doctorate.”

We apologize for this error.

